# P-1851. Influence of the COVID-19 Pandemic on Antimicrobial Resistance Patterns in Pediatric Group A *Streptococcus* Infections in Houston, TX

**DOI:** 10.1093/ofid/ofae631.2012

**Published:** 2025-01-29

**Authors:** Maria Gabriela Segura, Aya Aboulhosn, Misu A Sanson, Luis Vega, Sakina Chinwala, Lauren M Sommer, Jonathon C McNeil, Anthony R Flores

**Affiliations:** University of Texas, Houston, Texas; University of Texas, Houston, Texas; McGovern Medical School, Houston, TX; University of Texas, Houston, Texas; University of Texas, Houston, Texas; Baylor College of Medicine, Houston, Texas; Baylor College of Medicine, Houston, Texas; Vanderbilt University Medical Center, Nashville, TN

## Abstract

**Background:**

Group A *Streptococcus* (GAS) causes a large spectrum of disease, including invasive, pharyngeal and skin / soft tissue (SSTI)^1^. Penicillin remains the mainstay therapy but recently, resistance to second-line therapies has steadily increased, especially after COVID19, with macrolide-resistant GAS as an emerging threat^2^. Given recent surges of GAS associated with later stages of the pandemic,^4^ we sought to compare pre-pandemic (2013-2019) and pandemic (2020-2023) GAS epidemiology and antimicrobial resistance patterns.

**Figure 1:**
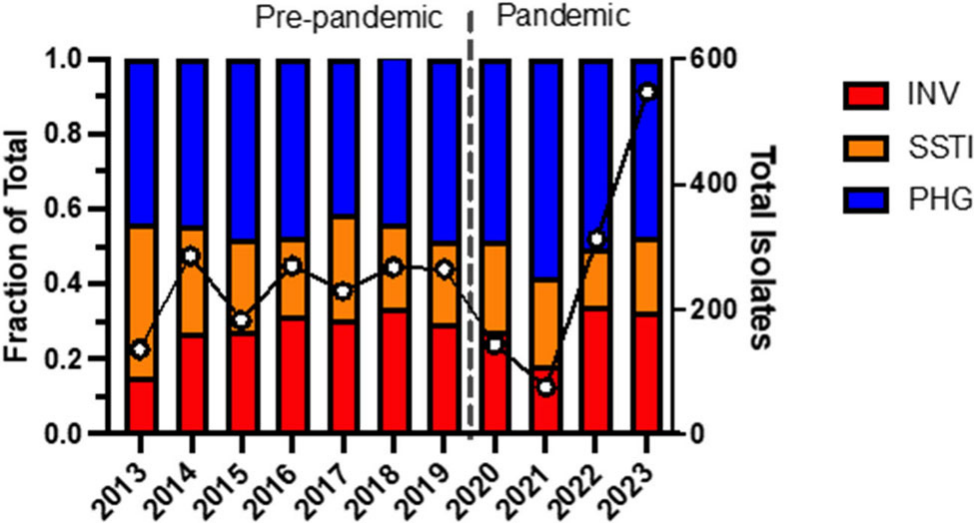
Total number of isolates and distribution of disease types per year.

**Methods:**

GAS isolates were collected from Texas Children’s Hospital laboratory from January 2013-December 2023 and grouped into pre-pandemic and pandemic periods. Disease type [invasive (INV), skin and soft tissue infection (SSTI), or pharyngeal (PHG)] was determined from the medical record. Isolates were grown, stocked and *emm* typed^5^. Resistance to tetracycline, erythromycin, and clindamycin was determined by disk diffusion. Intermediate and resistant strains were collectively considered non-susceptible (NS).

**Figure 2:**
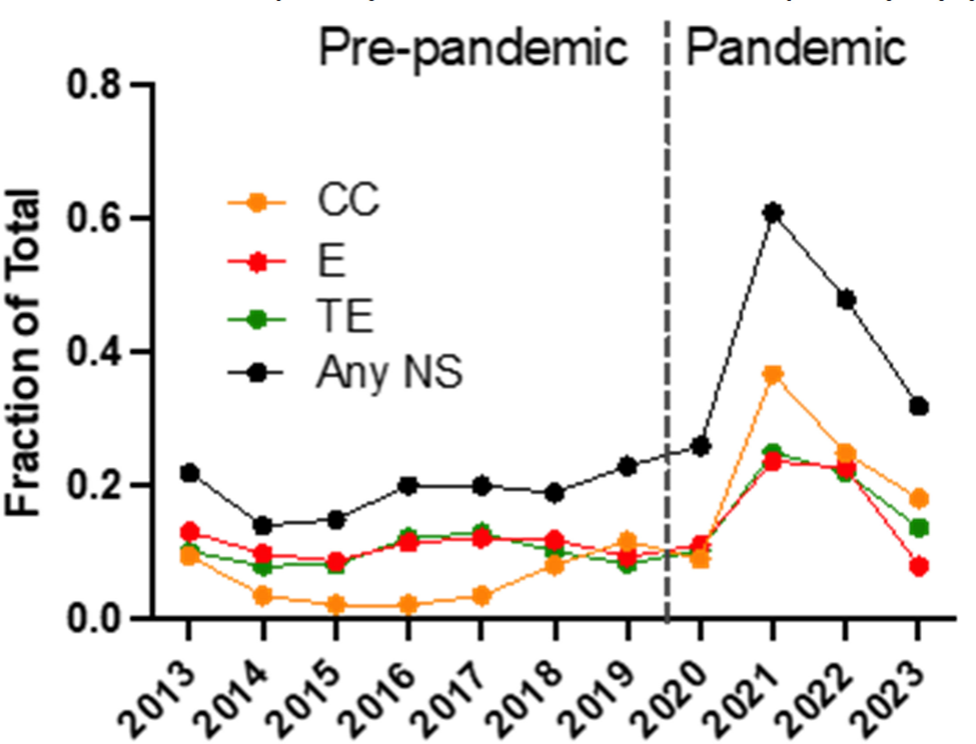
Frequency of antibiotic non-susceptibility by year

**Results:**

We analyzed 2,717 isolates (pre-pandemic n=1640; pandemic n=1077) and observed a similar distribution of disease types between the two periods (Figure 1). Distribution of *emm* types differed with *emm12* (1.7-fold) and *emm3* (1.6-fold) increasing significantly and *emm89* (0.6-fold), *emm4* (0.6-fold), *emm87* (0.4-fold) and *emm6* (0.3-fold) decreasing significantly. Overall NS to any antibiotic increased from 19% to 39% between the two periods (*P*< 0.0001). NS in the pandemic period was primarily driven by NS to clindamycin, increasing by >3-fold (6% vs 20%) (Figure 2). Among *emm* types showing NS to clindamycin pre vs pandemic, *emm28* increased 10-fold (3% vs 31%, *P*< 0.0001), *emm1* increased 5-fold (5% vs 24%, *P* =< 0.001), and *emm12* increased 2.6-fold (6% vs 16%, *P*< 0.001) (Figure 3). NS to any antibiotic increased independent of disease type between the two periods.

**Figure 3:**
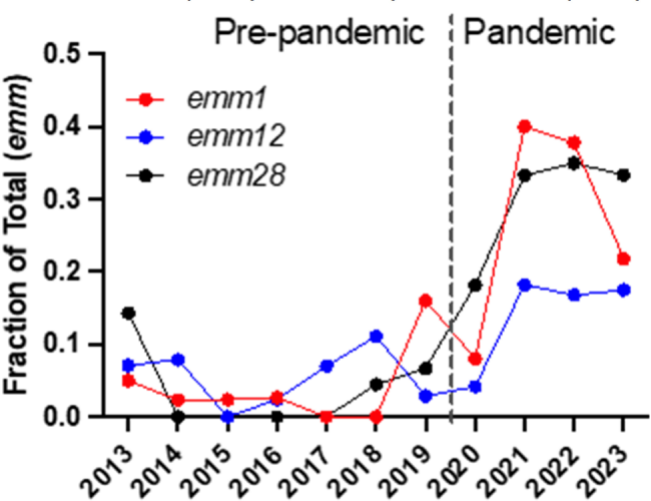
Frequency of clindamycin non-susceptibility by emm type

**Conclusion:**

NS increased significantly in pandemic period, mostly driven by NS to clindamycin. A small subset of *emm* types disproportionately contributed to clindamycin NS in pandemic period, although the mechanism remains unknown. Clinicians should be aware of the possible reduced effectiveness of clindamycin in pediatric GAS infections.

**Disclosures:**

**Jonathon C. McNeil, MD**, Nabriva: site investigator on clinical trial **Anthony R. Flores, MD, MPH, PhD**, GlycosBio, Inc: Grant/Research Support

